# Higher Dietary Se Intake Is Associated With the Risk of New-Onset Fracture: A National Longitudinal Study for 20 Years

**DOI:** 10.3389/fnut.2021.719147

**Published:** 2021-08-18

**Authors:** Yangchang Zhang, Mengliang Ye, Yong Zhao, Yang Xiong, Shisi Shen, Qiuhua Yu, Yanjun Lu, Zumin Shi, Xun Lei

**Affiliations:** ^1^School of Public Health and Management, Chongqing Medical University, Chongqing, China; ^2^Research Center for Medicine and Social Development, Chongqing Medical University, Chongqing, China; ^3^The Innovation Center for Social Risk Governance in Health, Chongqing Medical University, Chongqing, China; ^4^The West China Hospital, Sichuan University, Chengdu, China; ^5^The First School of Clinical Medicine, Chongqing Medical University, Chongqing, China; ^6^Human Nutrition Department, College of Health Sciences, QU Health, Qatar University, Doha, Qatar

**Keywords:** Se, fracture, CHNS, China, adults

## Abstract

**Background:** The association between dietary selenium (Se) intake and osteoporosis-related fractures remains inconsistent. We aimed to examine the dose relationship between Se intake and incident fracture among Chinese adults.

**Methods:** The dietary data were retrieved from the China Health and Nutrition Survey conducted between 1991 and 2011, and 17,150 participants aged above 20 were included. A 3-day, 24-h recall of food intake was performed to assess cumulative average dietary Se intake. The fracture was based on self-report in each survey between 1997 and 2011. The association between Se intake and fracture was tested by Cox regression, and the non-linear association was examined by restricted cubic splines (RCS).

**Results:** There were 976 fracture cases during a mean of 10.2 years follow-up. In a fully adjusted Cox model, across the quartiles of Se intake, the hazard ratios (HRs) for fracture were 1.07 (95% CI .86–1.33), 1 (reference), 1.25 (95% CI 1.02–1.53), and 1.33 (95% CI 1.07–1.65). RCS showed a parabolic association (*P* non-linear = 0.037) between Se and fracture for men as well as a U-shape dose-response (*P* non-linear = 0.04) between Se and fracture for subjects living in highly urbanized areas.

**Conclusion:** In conclusion, there is a non-linear association between selenium intake and fracture, with higher intake associated with increased risk. The shape of the association varies by gender and urbanization level.

## Introduction

Selenium, an essential trace element for human health, is involved in various physiological processes and function realization, such as immune response, anti-oxidation, anti-tumor, and thyroid hormone regulation (1). Previous studies have confirmed that in China, inadequate Se intake was linked to Keshan disease and Kashin-back disease (2). Meanwhile, other studies have demonstrated that excessive Se intake was also associated with chronic diseases, such as hypercholesterolemia, hypertension, and diabetes (3–6).

Grain, fish, and meat are major sources of Se intake for human beings (7). However, Se intake varies substantially by region. For example, the mean Se intake is 40 μg per day in Europe, while in the United States it is 93 μg per day for women and 134 μg per day for men (8, 9). According to the 2017 Reference Intake of Dietary Trace Elements issued by the National Health Commission of China, the estimated mean requirement (EAR), recommended nutrient intake (RNI), and tolerable upper limit intake (UL) of Se are 50, 60, and 400 μg, respectively (10). In addition, Se is unevenly distributed in the natural environment of China, about 72% of soil is Se-deficient (below.125 mg/kg), and the national average concentration of Se in soil is.239 mg/kg, ranging from.006 to 59.4 mg/kg (11, 12). The low-Se belt in the Chinese mainland (soil Se <0.2 mg/kg) sprawls from the northeast to the southwest, along with the Taihang and Qin Ling Mountains, Loess Plateau, and eastern part of the Tibetan Plateau (12). In contrast, the Se-rich belt (soil Se >20 mg/kg) spreads across Enshi in Hubei province, Ziyang in Shaanxi province, Shitai in Anhui province, and Yichun in Jiangxi province (13).

The relationship between Se intake and bone fracture has been explored in population-based studies (14–18). Se exists in the human body, mainly in the form of selenoprotein, for the maintenance of natural bone metabolism, proliferation, and differentiation; and the upkeep of homeostasis of extracellular matrix (ECM) (14) and bone mineral density (BMD) (15). Existing evidence suggests that the concentration of serum Se can affect the synthesis, fibril formation, and ultra-microstructure metabolism of cartilage collagen. Se deficiency is reported as an independent risk factor for abnormal cartilage collagen (16). Low Se in hair and plasma is related to BMD reduction (17, 18). However, inconsistent findings are reported on the association between Se intake and osteoporosis/fracture risk (17–22). A cross-sectional study of 7,407 middle-aged and older women in the United States, and a case-control study on 107 postmenopausal women in Turkey did not find any association between high Se intake and risk of osteoporosis and BMD (17, 19). A recent study on 1,365 Spanish adults (medium serum Se 84.9 μg/L at baseline) observed a U-shape dose-response relationship between serum Se and osteoporosis-related fracture (20). In contrast, a cross-sectional study in China argued that deficient dietary Se intake (<50 μg/day) is associated with higher prevalence of osteoporosis among 1,452 middle-aged and older people (21). The National Health and Nutrition Examination Survey (NHANES) among 2,983 American adults with relatively high Se intake (median Se intake of 101.5 μg/day), reports that higher Se intake was correlated with lower likelihood of bone fracture (22). The inconsistent findings of previous studies may be attributed to differences in study design, Se biomarkers, measuring instruments and methods for calculation of Se, and geographic regions and populations.

Few longitudinal studies have been conducted to examine the association between Se intake and risk of fracture. Although Se status is known for its antioxidant properties, the association between high Se intake and chronic disease remains unclear, probably attributed to its narrow safe window and other confounding factors (23, 24). In previous two national studies, higher Se intake decreased the risk of hypertension in north China but increased the risk of diabetes in the United States (23, 24). Furthermore, it has been pointed out that Se intake has a dose response to the incidence of diabetes and hypertension (4–6, 25–28). A cross-sectional study found that serum Se (referent point: 120 μg/L), Se hair (referent point: 8 μg/L), and Se nail (referent point: 6 μg/L) were related to blood pressure and hypertension in a U-shape trend (27). In a systematic review and meta-analysis, both low serum Se (<97.5 ug/L) and high serum Se (>132.5 ug/L) increased the risk of type 2 diabetes mellitus (T2DM) (26). Meanwhile, existing evidence suggests that patients with T2DM or hypertension have an increased risk of fracture, suggesting that chronic diseases may mediate the association between Se intake and risk of fracture (29, 30). Besides, Balvez-Fernandez et al. observed a U-shape curve for BMD and fracture associated with the increment of serum Se with a turning point of 105 μg/L (20).

Given this inconsistency, we hypothesized that a U-shape association exists between Se intake and fracture. Therefore, we aimed to investigate the association between dietary Se intake and fracture among Chinese adults aged 20 and above who participated in the China Health and Nutrition Survey (CHNS) between 1991 and 2011.

## Methods

### Study Sample

The China Health and Nutrition Survey is an ongoing longitudinal follow-up project conducted by the Carolina Population Center at the University of North Carolina at Chapel Hill and the National Institute for Nutrition and Health (NINH) at the Chinese Center for Disease Control and Prevention (CCDC), aiming to study the health and nutritional status of residents from different aspects, such as socio-demographics, economic development, public resources, and health indicators. More than 30,000 individuals from 7,200 households in 15 provinces were sampled by the multi-stage stratified cluster random sampling process. CHNS began in 1989, and so far, there have been 10 waves of surveys conducted (in 1989, 1991, 1993, 1997, 2000, 2004, 2006, 2009, 2011, and 2015), and respondents in waves 1991–2011 were adapted to examine the association between Se intake and fracture in this study because dietary data were available.

Respondents who participated in at least two waves after 1997 were included in the study; 25,252 candidates aged 20 and above were identified in the preliminary screening. Subsequently, respondents without record of food intake (*n* = 1123), abnormal in daily energy intake (males: >6000 kcal or <800 kcal; females: >4000 or <600 kcal) (*n* = 112) (31), in pregnancy or lactation (*n* = 168), with only once completed survey record or no specific fracture information (*n* = 6075) and having fracture at baseline (*n* = 714) were excluded. A total of 17,150 participants were finally eligible for the analyses, and there were 976 new fracture cases (Supplementary Figure 1). The survey was approved by the University of North Carolina (United States) and the National Institute of Nutrition and Food Safety (China). A written informed consent was obtained from every participant.

### Outcome Variable: Fracture

Self-reported history of fracture was collected by asking, “Have you ever had a fracture?” (32). In this study, the incident fracture was defined as a fracture that occurred in the follow-up period among those without a history of fracture at baseline.

### Exposure Variables: Se Intake

In the China Health and Nutrition Survey, a dietary assessment is based on the food intake of three consecutive 24-h recall at the individual level. A food inventory at the household level, which included all available stored and purchased foods, was weighted during the same 3-day period. The dietary intake of each respondent was recorded by asking each family member to report all the foods they ate at home and away from home over the past 24 h for 3 consecutive days. Trained interviewers collected the details of the intake, such as amounts and types of food, types of meal, and places of having food using standard forms. The 3 consecutive days were randomly allocated to 2 week days and a week end day across the week for each sampling unit. Household food consumption was estimated by weighing and comparing the changes in the inventory at the beginning and the end of the survey. Condiment consumption for each member was estimated by the ratio of individual to the whole household energy intake. All family storage and leftovers purchased from a grocery or picked from their gardens were also weighed and recorded. Preparation waste was estimated when exact weighing was not available. At the end of the survey, all the remaining foods were weighed again and recorded. The amount of food in each dish was calculated through the household inventory, reporting the proportion of consumption for each family member (33). The assessment of the mean daily Se intake (μg) of each respondent for each food item was derived from the dietary data module, and the food code was decoded from the Chinese Food Composition Table (CFCT). Three versions of CFCT were used to evaluate the intake of foods and nutrition in Chinese residents. To be specific, the 1981 CFCT was adopted for the waves of 1989, 1991, and 1993; the 1991 CFCT was available for 1997 and 2000; the 2002/2004 CFCT was adopted for 2004, 2006, 2009, and 2011. The CFCT took the region into consideration for the nutrient content of individual food items. Thus, the Se intake in the analysis was region-specific.

Because of the repeated measure design, we used three indicators of Se intake: baseline Se intake, cumulative average Se intake, and most recent Se intake. The use of the cumulative mean of Se intake aimed to reduce the variation within individuals and reflect long-term intake levels from 1991 to 2011. For example, when Se intake was *x, y*, and *z* in 1991, 1993, and 1997, the cumulative mean intake was *x* in 1991, (*x* + *y*)/2 in 1993, and (*x* + *y* + *z*)/3 in 1997. The most recent Se intake was measured in a specific wave.

### Covariates

Covariates, such as socio-demographics, health status, dietary patterns, and lifestyle factors, were collected with a structural questionnaire. Dietary foods were collapsed into 35 groups according to similar cooking styles and ingredients (34). Dietary patterns were constructed by factor analysis. The number of factors was determined according to the following rules: (1) eigenvalue >1; (2) scree plot; and (3) factor interpretability. Varimax rotation was used to help interpret the identified patterns. Two dietary patterns were identified and named “modern” and “traditional” dietary patterns (34). Physical activities were measured by consuming the metabolic equivalent of task (MET), which was calculated with the weighted mean of daily activities assessed with the Compendium of Physical Activities scale measuring the occupational, domestic, transportation, and leisure-time activities (35). An urbanization index was constructed and categorized into three levels (low, medium, and high), reflecting the living standards based on percentiles (32). Smoking status was categorized into non-smoker, ex-smokers, and current smokers. *Per capita*, annual household income was grouped as “low,” “medium,” and “high” based on tertiles. Body mass index (BMI) was used to assess obesity levels, and the cutoff point of overweight was 24 kg/m^2^ (36). Geographically, we defined Heilongjiang, Liaoning, Shandong, and Henan as the north; and Jiangsu, Hubei, Hunan, Guizhou, and Guangxi as the south (24).

Hypertension is defined as systolic blood pressure above 140 mmHg or diastolic blood pressure above 90 mmHg, or having clinically confirmed hypertension. Self-reported diabetes was recorded according to the question “Did you have a clinical diagnosis of diabetes?”

### Statistical Analyses

The continuous variables were described as mean ± standard deviation (SD), and the categorical variables were described as frequency and proportion (%). The categorical variables were analyzed by Chi-square test, and the continuous variables were tested by ANOVA or Kruskal–Wallis test. The multivariable Cox regression was used to assess the association between cumulative mean intake of dietary Se and incident fracture. The second quartile of Se intake (Q2) was set as the reference group. In sensitivity analyses, baseline and most recent dietary Se intakes were used in the Cox regression models instead of cumulative mean Se intake. A set of Cox regression models was built: Model 1- adjusting age, gender, and energy intake; Model 2- further adjusting smoking, alcohol drinking, income, urban, education, and physical activity; Model 3- further adjusting dietary patterns based on Model 2.

In the subgroup analyses, multiplicative interaction between dietary cumulative Se intake and covariates was tested by adding a cross-product term in the full multivariable Cox regression model, such as urbanization levels, sex, smoking, regions, and alcohol drinking. The RCS regression in the fully adjusted model was fitted with three knots (at 10, 50, and 90 percentiles) to examine the non-linear relationship between cumulative mean Se intake and incident fracture. We also conducted subgroup analyses by gender and urbanization levels.

All analyses were performed using Stata 16.1 (Stata Corporation, College Station, TX, United States). Statistical significance was considered when *P* < 0.05 (two-sided).

## Results

A total of 17,150 participants free of fracture at baseline were included in the analyses. Across the quartiles of Se intake, the mean (SD) of Se intake is 20 ± 5, 31.8 ± 2.9, 42.5 ± 3.6, and 71.2 ± 44.9, respectively, and the average Se intake for each evaluation wave is presented in Supplementary Figure 3. Table 1 shows that individuals in the fourth quartile of Se intake are more likely to (*P* < 0.001) have a higher intake of macronutrients (proteins, carbohydrates, and fats) and energy, to follow a modern dietary pattern (characterized by animal-based diet), to be younger (41.4 ± 14.4). The prevalence of overweight (29.2%) and current smoking (34.4%) was also higher among those with high Se intake. Men had a higher Se intake than women.

**Table 1:** Baseline sample characteristics by quartiles of selenium intake (*n* = 17,150).

**Factors**	**Q1**	**Q2**	**Q3**	**Q4**	***P*-value**
	***N* = 4,331**	***N* = 4,315**	***N* = 4,255**	***N* = 4,249**	
Selenium intake (ug/d), mean (SD)	20.0 (5.0)	31.8 (2.9)	42.5 (3.6)	71.2 (44.9)	<0.001
Energy intake (kcal/d), mean (SD)	1,793.9 (558.2)	2,059.1 (561.2)	2,281.0 (601.0)	2,540.4 (687.6)	<0.001
Fat intake (g/d), mean (SD)	51.4 (30.9)	62.8 (31.2)	72.0 (33.2)	86.1 (40.7)	<0.001
Protein intake (g/d), mean (SD)	48.0 (14.4)	61.6 (14.9)	72.5 (16.8)	88.8 (23.8)	<0.001
Carbohydrate intake (g/d), mean (SD)	283.1 (115.1)	308.5 (116.3)	331.3 (126.6)	345.8 (131.7)	<0.001
Traditional southern dietary pattern score, mean (SD)	0.1 (0.7)	−0.0 (0.9)	−0.0 (1.0)	0.1 (1.3)	<0.001
Modern dietary pattern score, mean (SD)	−0.5 (0.6)	−0.3 (0.8)	0.0 (0.9)	0.6 (1.3)	<0.001
Age (years), mean (SD)	44.0 (16.3)	42.7 (15.4)	41.9 (14.8)	41.1 (14.4)	<0.001
BMI (kg/m2), mean (SD)	22.4 (3.3)	22.7 (3.3)	22.9 (3.3)	23.3 (3.4)	<0.001
Overweight (%)	19.1%	23.3%	24.9%	29.2%	<0.001
Sex (%)					<0.001
Men	37.3%	43.2%	52.5%	59.6%	
Women	62.7%	56.8%	47.5%	40.4%	
Income (%)					<0.001
Low	38.7%	28.7%	24.4%	20.0%	
Medium	33.5%	35.4%	34.2%	30.3%	
High	27.8%	35.8%	41.4%	49.7%	
Education (%)					<0.001
Low	52.8%	40.1%	33.7%	26.6%	
Medium	29.2%	33.6%	34.9%	35.1%	
High	18.1%	26.3%	31.5%	38.3%	
Diabetes (baseline in 2009 only, %) *n* = 1228; 214, 274, 409, 2038	7.5%	8.4%	8.2%	9.5%	0.83
Self-reported diabetes (%)	1.6%	1.8%	2.0%	2.9%	<0.001
Hypertension (%)	15.9%	16.3%	16.7%	17.5%	0.25
Urbanization (%)					<0.001
Low	43.5%	30.3%	24.4%	19.3%	
Medium	27.6%	29.9%	27.8%	25.7%	
High	28.9%	39.7%	47.7%	55.0%	
Smoking (%)					<0.001
Non-smoker	73.6%	70.6%	66.2%	63.2%	
Ex-smokers	2.0%	1.8%	1.9%	2.4%	
Current smokers	24.4%	27.6%	32.0%	34.4%	
7–9 h	76.1%	79.8%	81.7%	79.7%	
< = 6 h	11.5%	10.1%	8.8%	9.7%	
> = 10 h	12.4%	10.1%	9.5%	10.6%	
Physical activity (MET-hrs/week), mean (SD)	143.8 (118.2)	135.7 (113.0)	129.4 (108.0)	128.5 (108.7)	<0.001
Survey year (%)					<0.001
1997	49.1%	47.7%	46.1%	37.3%	
2000	14.3%	15.0%	13.3%	13.7%	
2004	9.1%	7.8%	8.3%	11.2%	
2006	3.9%	4.7%	5.1%	6.5%	
2009	6.6%	8.0%	9.5%	11.8%	
2011	16.9%	16.7%	17.8%	19.6%	

*BMI, body mass index; MET, metabolic equivalent; h, hours*.

A total of 976 incident fractures occurred during the study period, and the median follow-up time was 9.1 years (147,770 person-year). The incidence rates were 5.8, 6, 7, and 7.7 per 1,000 person-years across the quartiles of cumulative Se intake. The cumulative Se intake was positively associated with fracture after adjusting for age and gender. Compared with the second quartile of Se intake, the hazard ratios (HRs) and 95% confidence intervals (CIs) for the fracture were 1.22 (95% CI: 1–1.5) in the third quartile and 1.25 (95% CI: 1.01–1.54) in the fourth quartile when sociodemographic and lifestyle factors were adjusted (*P*-trend <0.05). The association became slightly stronger when dietary patterns were adjusted, with HRs of 1.25 (95% CI: 1.02–1.53) in the third quartile and 1.33 (95% CI: 1.07–1.65) in the fourth quartile. Compared with model 3, the association stayed robust steadily when diabetes and hypertension were adjusted in model 4, with HR of 1.24 (95% CI:1.03–1.49) in the third quartile and 1.3 (95% CI: 1.07–1.58) in the fourth quartile (Table 2). A positive association between high Se intake and fracture was also found when we used baseline Se intake and most recent Se intake. Supplementary Figure 2 shows the association between sociodemographic factors and fracture. Age, high income, urbanization levels, and overweight were positively associated but education was inversely associated with the risk of fracture.

**Table 2:** Hazard ratio (95%CI) for incident fracture by quartiles of selenium intake.

**Quartiles of selenium intake**	**Q1**	**Q2**	**Q3**	**Q4**	***P* for trend**
**A. Cumulative average selenium intake**					
Cases	226	219	253	278	
Person-years	38,732	36,754	36,123	36,161	
Incident rate (per 1000)	5.8	6.0	7.0	7.7	
Model 1	0.99 (0.82–1.20)	1.00	1.18 (0.99–1.42)	1.36 (1.13–1.63)	0
Model 2	1.09 (0.88–1.36)	1.00	1.22 (1.00–1.50)	1.25 (1.01–1.54)	0.092
Model 3	1.07 (0.86–1.33)	1.00	1.25 (1.02–1.53)	1.33 (1.07–1.65)	0.021
Model 4	1.02 (0.83–1.24)	1.00	1.24 (1.03–1.49)	1.30 (1.07–1.58)	0.005
**B. Baseline selenium intake**					
Cases	217	257	258	244	
Person-years	40,567	38,197	35,992	33,014	
Incident rate (per 1000)	5.3	6.7	7.2	7.4	
Model 1	0.76 (0.64–0.91)	1.00	1.08 (0.91–1.29)	1.13 (0.95–1.35)	0
Model 2	0.84 (0.68–1.03)	1.00	1.09 (0.89–1.32)	1.06 (0.87–1.30)	0.023
Model 3	0.82 (0.67–1.01)	1.00	1.11 (0.91–1.34)	1.10 (0.89–1.35)	0.006
Model 4	0.79 (0.65–0.95)	1.00	1.05 (0.88–1.26)	1.10 (0.92–1.32)	0.001
**C. Most recent selenium intake**					
Cases	235	221	252	268	
Person-years	38,194	37,183	36,908	35,486	
Incident rate (per 1000)	6.2	5.9	6.8	7.6	
Model 1	0.99 (0.82–1.20)	1.00	1.17 (0.98–1.41)	1.29 (1.07–1.55)	0.003
Model 2	1.14 (0.92–1.42)	1.00	1.18 (0.96–1.46)	1.21 (0.98–1.50)	0.335
Model 3	1.11 (0.90–1.38)	1.00	1.21 (0.98–1.49)	1.29 (1.03–1.62)	0.112
Model 4	1.12 (0.92–1.36)	1.00	1.19 (0.99–1.44)	1.30 (1.06–1.60)	0.074

*Model 1 adjusted age, gender, and energy intake*.

*Model 2 further adjusted intake of fat, smoking, alcohol drinking, income, urban, education, and physical activities*.

*Model 3 further adjusted dietary patterns ^20^(traditional south pattern represented high intake of rice, pork, and vegetables, and low intake of wheat; modern dietary pattern represented high intake of fruit, soy milk, egg, milk, and deep-fried food)*.

*Model 4 further adjusted for self-reported diabetes and hypertension*.

*All the adjusted variables were treated as time-varying covariates*.

In the subgroup analyses (Table 3), we did not observe any significant effects of sex, alcohol drinking, and smoking on the association between cumulative Se intake and fracture, while living at a high urbanization level was detected to exacerbate the association (*P*-interaction = 0.013). Among those living in a highly urbanized area, high Se intake was associated with an increased risk of fracture (HR: 1.76, 95% CI: 1.26–2.46) compared with the second quartile of Se intake.

**Table 3:** Association between quartiles of cumulative selenium intake and fracture stratified by residence, gender, hypertension, region, and drinking.

**Factors**	**Q1**	**Q2**	**Q3**	**Q4**	***P* for interaction**
**Urbanization levels**					0.013
Low	0.74 (0.50–1.09)	1.00	0.83 (0.54–1.30)	1.31 (0.84–2.05)	
Medium	1.00 (0.69–1.44)	1.00	1.40 (1.00–1.97)	0.96 (0.65–1.42)	
High	1.59 (1.09–2.31)	1.00	1.47 (1.05–2.04)	1.76 (1.26–2.46)	
**Sex**					0.644
Men	0.92 (0.66–1.28)	1.00	1.15 (0.87–1.51)	1.28 (0.97–1.70)	
Women	1.19 (0.88–1.60)	1.00	1.37 (1.01–1.86)	1.37 (0.98–1.92)	
**Smoking**					
No	1.23 (0.95–1.60)	1.00	1.35 (1.04–1.76)	1.51 (1.15–1.99)	0.338
Yes	0.74 (0.49–1.11)	1.00	1.09 (0.78–1.51)	1.07 (0.75–1.52)	
**Alcohol drinking**					
No	1.07 (0.83–1.39)	1.00	1.12 (0.86–1.46)	1.19 (0.89–1.58)	0.204
Yes	0.99 (0.65–1.48)	1.00	1.46 (1.05–2.04)	1.55 (1.10–2.18)	
**Region**					0.788
North	0.81 (0.56–1.16)	1.00	1.17 (0.85–1.61)	1.30 (0.94–1.80)	
South	1.03 (0.79–1.33)	1.00	1.22 (0.95–1.56)	1.07 (0.81–1.42)	

*Models adjusted for the same covariates as model 3 in Table 2. Stratification variables were not adjusted in the corresponding models*.

**Figure 1:**
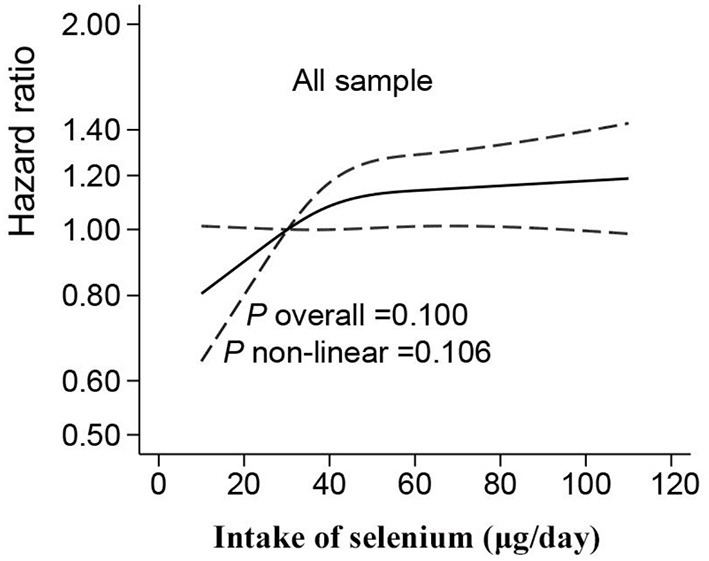
Hazard ratios for fracture according to selenium intake in all the samples. Data were fitted by Cox proportional hazard regression. The solid line represents estimates (hazard ratio) using restricted cubic splines, and dash lines represent 95% CI. The models adjusted the same covariates with model 3 in Table 2.

**Figure 2:**
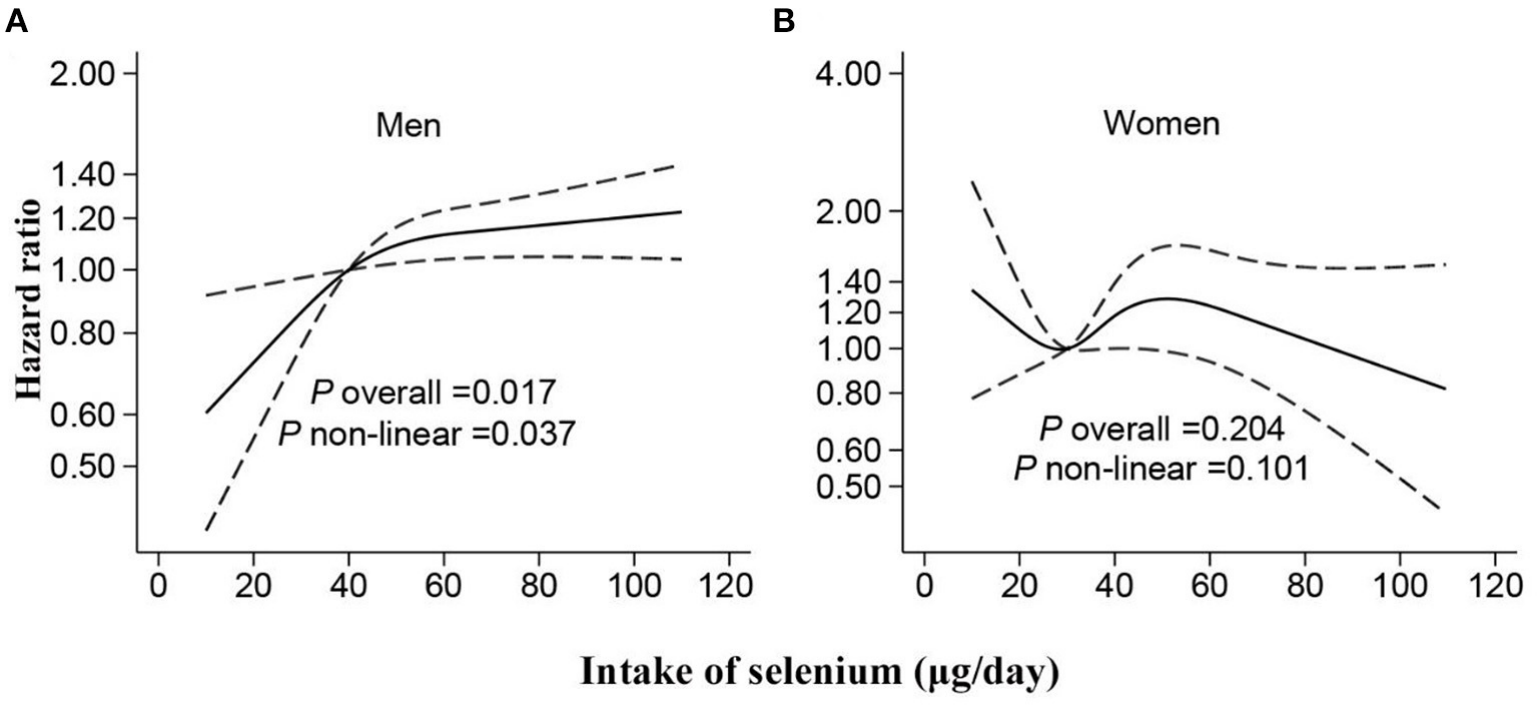
Hazard ratios for fracture according to selenium intake stratified by gender [**(A)** for men; **(B)** for women]. Data were fitted by Cox proportional hazard regression. The solid line represents estimates (hazard ratio) by restricted cubic splines, and the dash lines represent 95% CI. The models adjusted the same covariates with model 3 in Table 2.

**Figure 3:**
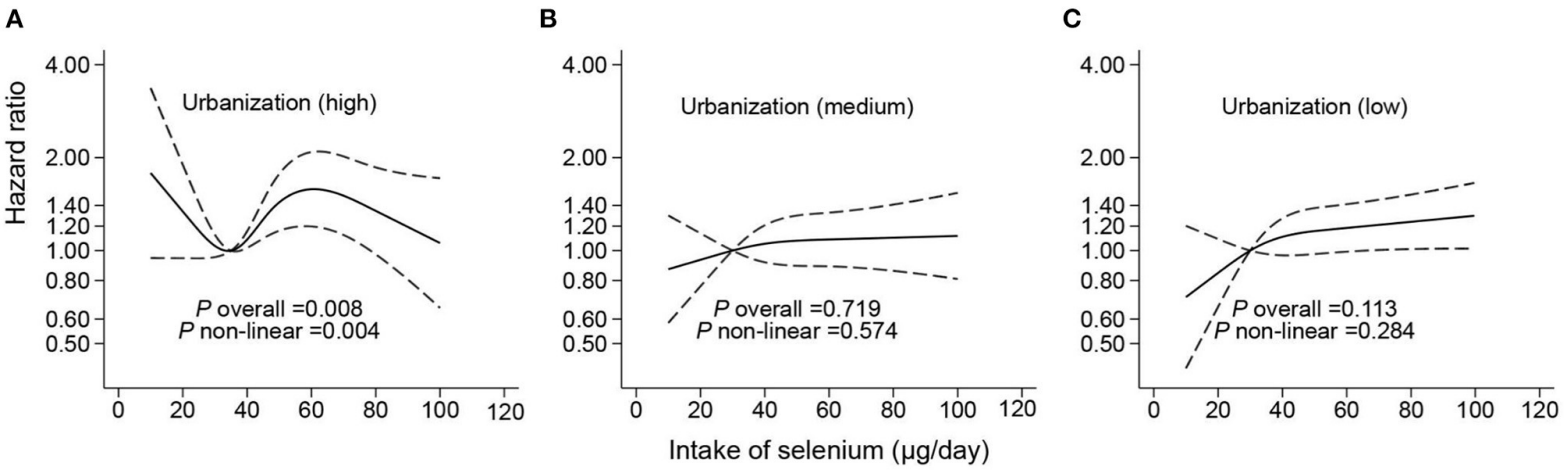
Hazard ratios for fracture according to selenium intake stratified by urbanization level [**(A)** for high urbanization, **(B)** for medium urbanization, and **(C)** for low urbanization]. Data were fitted by Cox proportional hazard regression. The solid line represents estimates (hazard ratio) using restricted cubic splines, and the dash lines represent 95% CI. The models adjusted the same covariates with model 3 in Table 2.

A non-linear association between Se intake and fracture was found in restricted cubic splines analyses. Overall, no significant associations were determined in the RCS in the whole or in women (Figures 1, 2B). However, there was a significant non-linear association for men (Figure 2A). In addition, a U-shape association was found between Se intake and fracture for participants living in highly urbanized areas (*P*-non-linearity = 0.004), with the lowest risk around the intake of 30 μg/day (Figure 3A). Additional complete information on restricted cubic splines analyses was performed in Figure 2B and Figure 3B as well as Figure 3C.

## Discussion

In this population-based study with 17,150 Chinese adults, there was a positive association between high Se intake and fracture, independent of sociodemographic and lifestyle factors, such as dietary patterns. The restricted cubic splines showed that the association was non-linear, particularly for men and subjects living in highly urbanized areas. In addition, the average Se intake ranges from 41.5 μg/day in 1991 to 41.9 μg/day in 2011, which has generally been rising since 1997 (Supplementary Figure 3). However, the overall Se intake is lower than China EAR (50 μg/day). Meanwhile, the Se intake of adults in China was relatively insufficient compared with that of adults in other countries (22, 37–39). In China, the median dietary intake is 40.8 μg/day for men and 39.5 μg/day for women (38). In the United States, the geometric means dietary Se intake was 101.5 μg/day according to a study (22), and the median dietary Se intake varied between 79–99 μg/day in another research (34). In Europe, the median of dietary Se intake was 94.3 μg/day (37).

The association between Se status and fracture is inconsistent. A longitudinal study in Spain showed that participants with higher Se intake had a significant risk of fracture (20). In contrast, a cross-sectional study in the United States found an inverse correlation between blood, serum, and dietary Se with the occurrence of fracture (22), which was subsequently confirmed by another two studies performed in the United States and China (38, 39). Nevertheless, no association was found between Serum Se and fracture subjects from five European cities (37). This study found that dietary Se intake was positively associated with fracture, and the association was strengthened after dietary patterns were adjusted, which was consistent with the previously published study in Spain (20). In the longitudinal study in Hortega, HRs for fracture across the tertiles of plasma Se were 1, 1.09 (95% CI: 55–2.16), 1.67 (95% CI: 91–3.04), and the HR of fracture for 80th percentiles of plasma Se distribution was 2.25 (95% CI: 1.13–4.49) compared with the 20th in a model that adjusted for age, sex, BMI, education, physical activity, urine cotinine, glomerular filtration, smoking, and alcohol drinking (20). However, negative associations between dietary Se intake and fracture were detected by the National Health and Nutrition Examination Surveys (NHANES) in the United States and a case-control study in China (22, 38). The possible reasons might be that the NHANES was a cross-sectional study and had a high baseline Se intake (mean 101.5 μg/day) and that several bone parameters were applied, such as total spine and femur BMD and Fracture Risk Assessment Tool (FRAX) scores and history of bone fractures (22). The limitations of the NHANES study included its cross-sectional study design and lack of adjustment for physical activity in the analysis. Similarly, with 726 pairs, the duration of exposure was difficult to confirm, and recall bias might also be introduced in the Chinese case-control study. Moreover, measuring errors of antioxidants might also lead to over-or underestimates of Se intake (38). A 7-year multi-center study across five European cities indicated that high serum Se was not associated with hip fracture incidence among postmenopausal women (37), which was opposite to the findings of this study. It is probably because only few fracture cases (31 vertebral fractures, 80 non-vertebral fractures, seven hip fractures) were reported in the 2,374 healthy participants.

Several factors may explain the positive association between dietary Se intake and fracture. First, long-term high Se intake is associated with an increased prevalence of diabetes (26, 28). It has been shown that the use of hypoglycemic drug is associated with a fracture in patients with type 2 diabetes (40), and that among medications, the use of thiazolidinediones and insulin has a greater impact (41). Besides, hyperglycemia may also result in the decline of osteogenic differentiation and bone turnover, reducing the bone quality and muscle mass of patients (42). Second, hypertension may have an increasing arteriosclerosis index, positively related to BMD decrement and fracture occurrence (43). Thirdly, unlike the findings in Hortega, the association between Se intake and fracture risk in this study is found to be independent of dietary patterns, suggesting potential mechanisms other than food preference. Se is a well-known trace element with a narrow gap between the safe and toxic levels (44). It has been shown that a dietary Se intake of over 55 μg/day cannot improve selenoprotein synthesis or activity in Se-replete subjects (45, 46). In addition, a previous study has argued that oxidative stress biomarkers (GSSG/GSH and MDA) and serum Se concentration have an inverse effect when serum Se is below 110 μg/L, and that GPx1 reaches the saturation status (47). It has been verified that oxidative stress (OS) biomarkers might reach a plateau value at this point (48). The findings of this study added further evidence to the view that higher dietary Se intake, exceeding the threshold required for maximum antioxidant protection by GPx1, may reverse the effect of selenoprotein osteoblast proliferation, differentiation, and osteoclast activity (49). Nevertheless, there is no universal agreement on the optimal intake of Se with the saturation of selenoproteins, suggesting that further epidemiological and clinical evidence is needed.

In this study, the fracture risk showed a sharply increasing curve when cumulative Se intake was lower than 30 μg/day for men, and the increment of risk turned to slow down when it exceeded 30 μg/day. However, a dose-response relationship was not detected in women, which probably resulted from the sex difference in Se storage and metabolism (50–53). Laboratory experiments on rodent models also showed that a higher concentration of Se in serum and red blood cell (RBC) was found in female rats compared with male rats after being fed with Se supplementation (52, 53). In addition, a clinical trial was performed with healthy subjects at a high Se baseline, and four different doses of selenomethionine were provided (50). The overall serum Se of all the subjects was heightened after a year of intervention, but the concentration of selenoprotein remained invariant, which suggested that a high Se supplement might not enhance the activity of saturated selenoprotein (50). Furthermore, another study found that women had higher urinary Se excretion than men when receiving equal doses of selenomethionine, suggesting that sexual dimorphism exists in Se intake and metabolism in the human body (51). In addition, the hormonal status will also have an influence. A high estrogen level for premenopausal women may protect them from osteoporosis and fracture, while a low testosterone level may cause loss of bone mass (54, 55).

A U-shape relationship between dietary Se intake and fracture risk was found among participants living in highly urbanized areas. It could be attributed to the interaction with air pollution. In highly urbanized regions, the environmental concentration of Arsenic (As), cadmium (Cd), lead (Pb), and black carbon (BC) is high (56). PM2.5 is the mass of particles per cubic meter in the air, with a size (diameter) generally <2.5 micrometers (μm), and is well-known as delicate particulate matter (2.5 micrometers is one 400th of a millimeter) (57). Studies have found that PM2.5 and BC were positively associated with an osteoporosis-related fracture (58). In addition, Se was also reported to have a strong affinity with heavy metals with which Se would combine to form non-toxic mental-bound selenoproteins and then excrete them from the body (59). In the second half of the curve, an exponential increment was found among those with high urbanization levels. The possible cause is that the western dietary pattern, containing high fat and protein from animal sources, is more prevalent among urban residents. Besides, residents in highly urbanized areas may have a higher prevalence of obesity and T2DM than the rural ones (60). Therefore, the combined effects of T2DM and obesity on fracture could be the mediator on the path from high Se intake to fracture occurrence in highly urbanized areas. Some studies found a pooled effect of diabetes and obesity on bone turnover and fracture in Finland and Canada (61, 62). Additionally, obesity may increase fracture risk independent from chronic disease, because increasing the bone burden and unbalanced buffer capacity of bone tissue provides an external impact (63).

Several strengths of this study need to be highlighted. First, most existing studies are focused on the association between most current dietary Se intake and fracture occurrence, so only few studies have explored the long-term effects. We included measures of 3-day dietary intake repetitively over 20 years, and used cumulative Se intake to reduce the error caused by the grand mean of single 3-day foods measures. Thus, Se intake may reflect long-term intake. Second, we examined the dose-response relationship between Se intake and fracture in various subgroups. Through this approach, we examined interactions and non-linear association. It may help to explore the threshold and saturation values of Se intake for the development of a precise nutrition intervention.

Several limitations of this study should also be considered. First, the use of a 3-day dietary intake survey may not adequately characterize long-term dietary Se intake. Second, the information collected from the respondents may suffer from recall bias on the history of fractures and other self-report variables. Although BMD and Fracture Risk Assessment Tool (FRAX) scores were both indicators of bone health, participants of CHNS were not examined for these indicators. Third, we did not have biomarkers of Se (e.g., hair Se, serum Se). Future studies will be expected to examine the association between the biomarkers of Se and fracture.

## Conclusion

There is a non-linear association between Se intake and fracture. High Se intake increases the risk of fracture among Chinese adults. Along with the subsequent rise in Se intake, there is a parabolic increment of fracture risk in men and a U-shape fracture risk among people who live in highly urbanized areas. Further research is needed to make evidence-based policies and guidelines regarding Se intake for the promotion of bone health.

## Data Availability Statement

The raw data supporting the conclusions of this article will be made available by the authors, without undue reservation.

## Ethics Statement

The studies involving human participants were reviewed and approved by The Institutional Review Board of the University of North Carolina at Chapel Hill and the National Institute for Nutrition and Health, Chinese Center for Disease Control and Prevention. The patients/participants provided their written informed consent to participate in this study.

## Author Contributions

YZhang and ZS contributed to the conception, analysis, and interpretation of data, drafted the report, and received the final version for publication. MY, YZhao, QY, SS, YL, and XL contributed to the analysis and interpretation of data, commented on the report, revised the manuscript, and approved the final version for submission. All authors read and approved the published version of the manuscript.

## Conflict of Interest

The authors declare that the research was conducted in the absence of any commercial or financial relationships that could be construed as a potential conflict of interest.

## Publisher's Note

All claims expressed in this article are solely those of the authors and do not necessarily represent those of their affiliated organizations, or those of the publisher, the editors and the reviewers. Any product that may be evaluated in this article, or claim that may be made by its manufacturer, is not guaranteed or endorsed by the publisher.

## Abbreviations

CHNS: china health and nutrition survey
BMI: body mass index
BMD: bone mass density
Se: selenium
RCS: restricted cubic splines
HR: hazard ratio
CI: confidence interval
NHANES: national health and nutrition examination surveys
MET: metabolic equivalent of task
CDC: centers for disease control.
