# Supplementary material for: Higher Dietary Se Intake Is Associated With the Risk of New-Onset Fracture: A National Longitudinal Study for 20 Years
Source: Front Nutr. 2021 Aug 18;8:719147. doi: 10.3389/fnut.2021.719147 (PMC8416262; doi:10.3389/fnut.2021.719147)

Supplementary Material

#
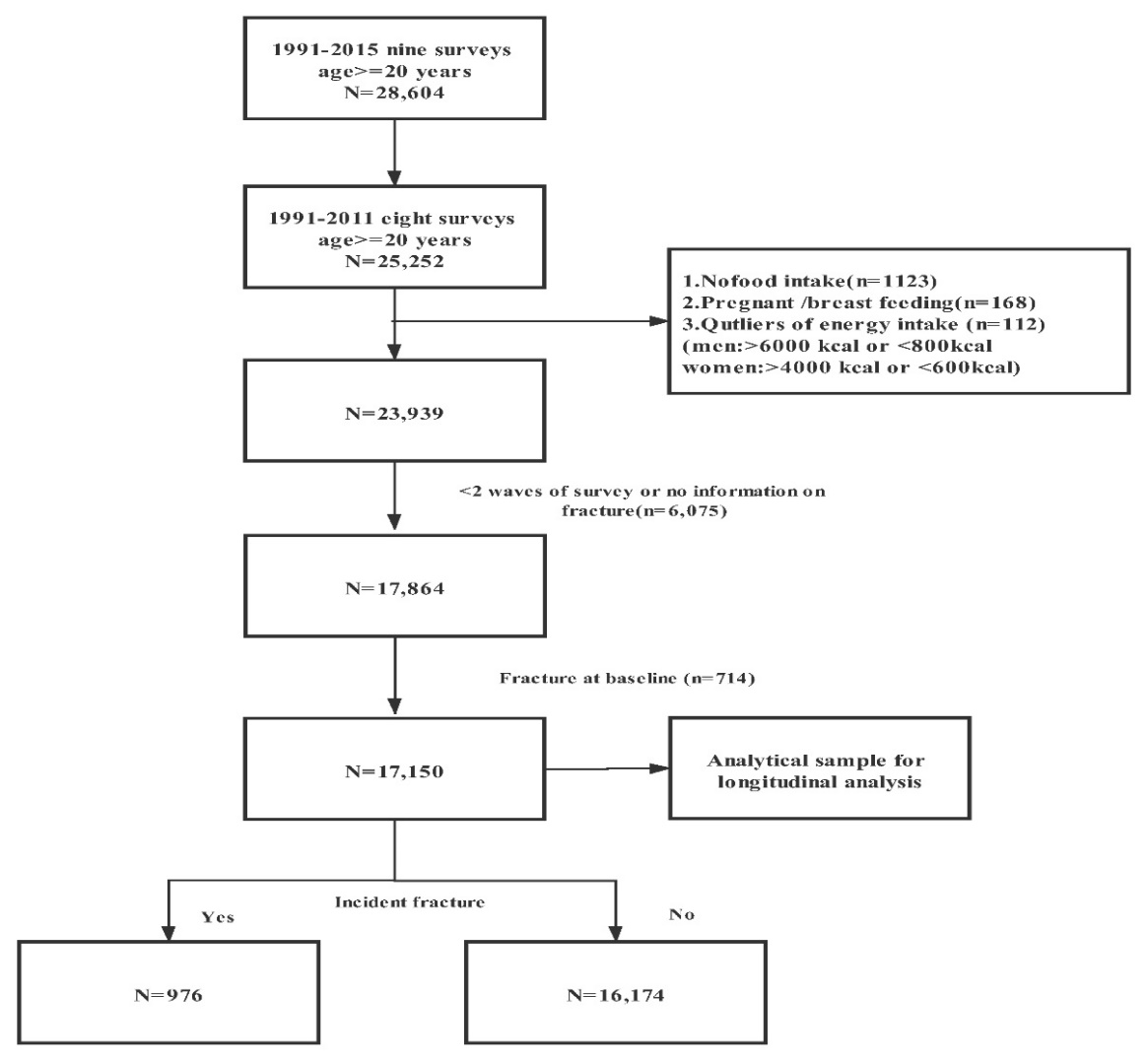
Supplementary Figure 1 Sample flow chart.

**Supplementary Figure 2.** The association between sociodemographic factors and fracture during 1991 to 2011.


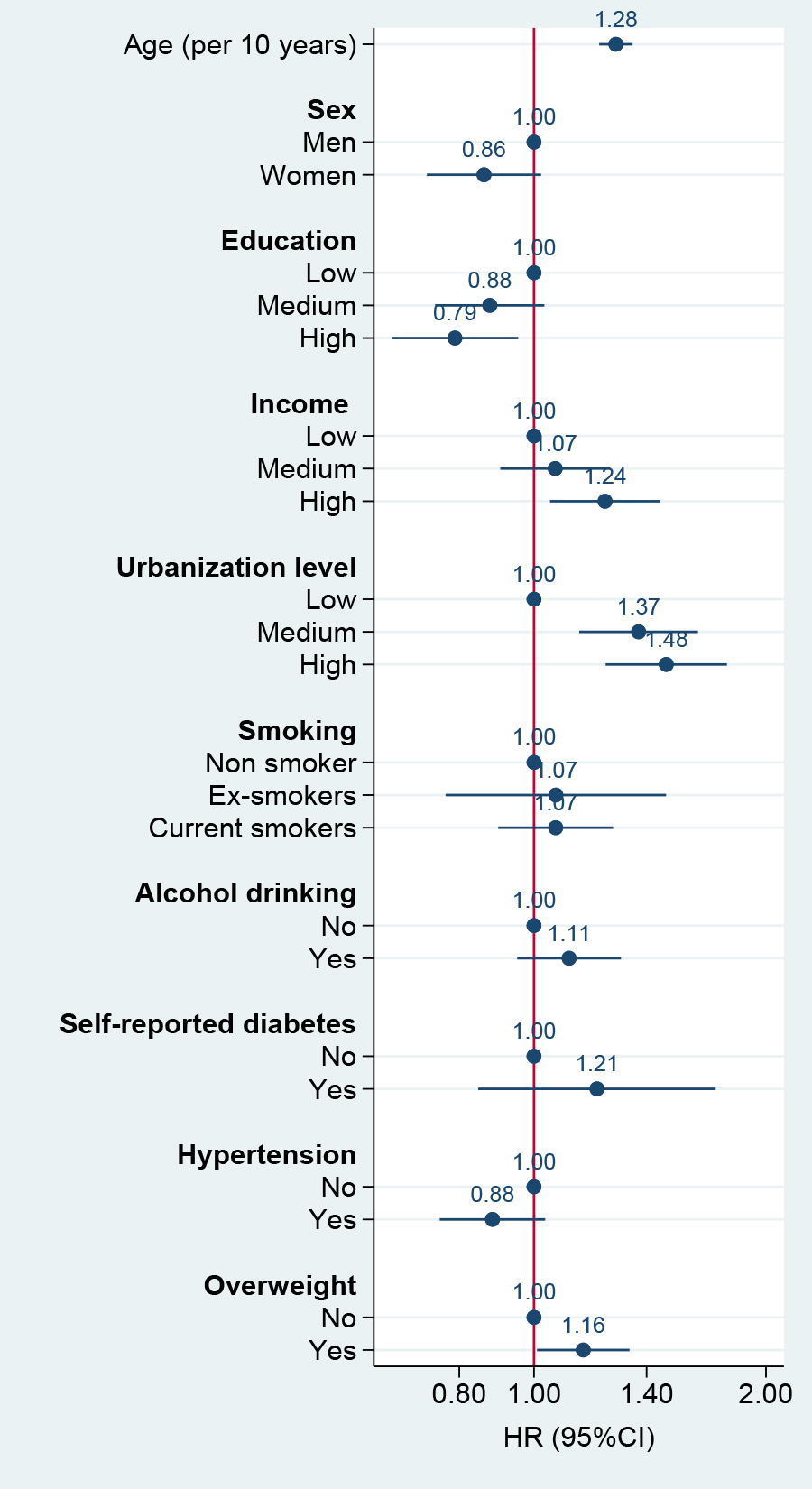


**Supplementary Figure 3.** Mean Se intake (μg/d) and 95% CI among adults aged 20 years and above during 1991-2011.


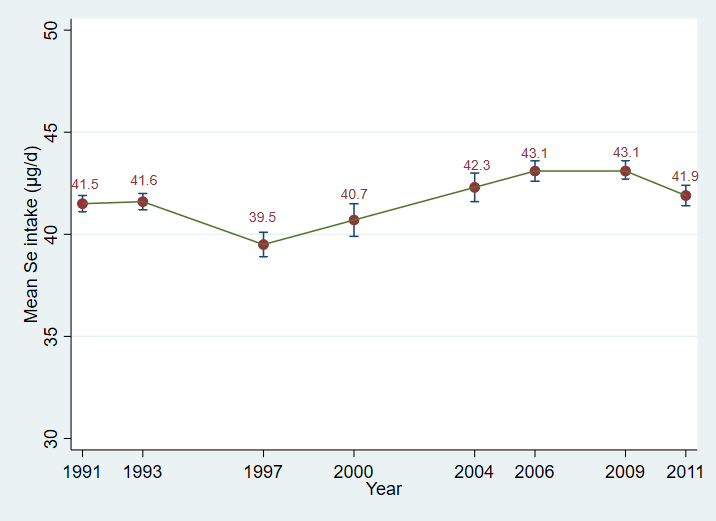

Supplement: Supplementary file 1 [file Data_Sheet_1.docx]
